# Plasma Enabled Fe_2_O_3_/Fe_3_O_4_ Nano-aggregates Anchored on Nitrogen-doped Graphene as Anode for Sodium-Ion Batteries

**DOI:** 10.3390/nano10040782

**Published:** 2020-04-18

**Authors:** Qianqian Wang, Yujie Ma, Li Liu, Shuyue Yao, Wenjie Wu, Zhongyue Wang, Peng Lv, Jiajin Zheng, Kehan Yu, Wei Wei, Kostya (Ken) Ostrikov

**Affiliations:** 1 School of Electronic and Optical Engineering Nanjing University of Posts and Telecommunications, Nanjing 210023, China; wqq1242103532@163.com (Q.W.); 1017030916@njupt.edu.cn (Y.M.); a884205500701@icloud.com (L.L.); ysynn0916@163.com (S.Y.); wuvenjie@163.com (W.W.); zywang@njupt.edu.cn (Z.W.); lvp@njupt.edu.cn (P.L.); zhengjj@njupt.edu.cn (J.Z.); weiwei@njupt.edu.cn (W.W.); 2 School of Chemistry and Physics, Queensland University of Technology, Brisbane QLD 4000, Australia; kostya.ostrikov@qut.edu.au; 3 CSIRO-QUT Joint Sustainable Processes and Devices Laboratory P.O. Box 218, Lindfield NSW 2070, Australia

**Keywords:** iron oxide, graphene, phase boundary, plasma, sodium-ion battery

## Abstract

Low electrical conductivity severely limits the application of Fe_2_O_3_ in lithium- and sodium-ion batteries. In respect of this, we design and fabricate Fe_2_O_3_/Fe_3_O_4_ nano-aggregates anchored on nitrogen-doped graphene as an anode for sodium-ion batteries with the assistance of microwave plasma. The highly conductive Fe_3_O_4_ in the composite can function as a highway of electron transport, and the voids and phase boundaries in the Fe_2_O_3_/Fe_3_O_4_ heterostructure facilitate Na^+^ ion diffusion into the nano-aggregates. Furthermore, the Fe–O–C bonds between the nano-aggregates and graphene not only stabilize the structural integrity, but also enhance the charge transfer. Consequently, the Fe_2_O_3_/Fe_3_O_4_/NG anode exhibits specific capacity up to 362 mAh g^−1^ at 100 mA g^−1^, excellent rate capability, and stable long-term cycling performance. This multi-component-based heterostructure design can be used in anode materials for lithium- and sodium-ion batteries, and potential opens a new path for energy storage electrodes.

## 1. Introduction

Insufficient lithium resources will seriously threaten the availability of future lithium-ion batteries (LIB). Owing to a similar working mechanism to LIBs, abundance, lower price, and environmental friendliness, sodium-ion batteries (SIBs) have gradually become a hotspot for energy storage [1,2,3,4]. Advances in SIB technology rely on the selection of suitable electrode materials to accommodate, insert, and extract Na^+^ ions with a larger radius than Li^+^ [5,6].

Among many metal oxides, the theoretical capacity of Fe_2_O_3_ is as high as ~1007 mAh g^−1^, and its environmental friendliness, low cost, and abundant resources make it an ideal anode material for SIBs [7]. However, Fe_2_O_3_ undergoes a large volume expansion (200%) during charge-discharge, resulting in electrode pulverization and deteriorated electrical contact problems [8,9]. On the other hand, the low electrical conductivity of Fe_2_O_3_ (10^−14^ S cm^−1^) and low ionic diffusivity limits the rate performance of batteries [10,11]. Common strategies are to design nanosized iron oxide, and to use conductive additives, such as graphene [9,12,13]. Another iron-based oxide, Fe_3_O_4_, has a slightly lower specific capacity (926 mAh g^−1^) and much higher electrical conductivity (10^2^~10^3^ S cm^−1^) than Fe_2_O_3_ [14]. Rationally compounding Fe_2_O_3_ and Fe_3_O_4_ may improve the conductivity, facilitate the redox charge transfer, and thus the rate performance. As has been demonstrated in supercapacitors, the conductive Fe_3_O_4_ and capacitive Fe_2_O_3_ synergistically produced excellent rate capability and cycling stability [15,16,17]. Recently, α-Fe_2_O_3_/Fe_3_O_4_ composite, Fe_2_O_3_/Fe_3_O_4_/FeCO_3_ composite, and porous Fe_2_O_3_/Fe_3_O_4_@carbon have also been applied in LIBs and exhibited improved electrochemical performance in comparison with the Fe_2_O_3_ electrodes [18,19,20]. In these studies, the Fe_3_O_4_ plays the role of electron transport pathway, while the porous structure can facilitate fast ion transport. It is even claimed that the hetero-interfaces between the oxide components may provide an enhanced inner electric field which can assist the electron transfer and Li^+^ diffusion [19].

In this work, we designed Fe_2_O_3_/Fe_3_O_4_ nano-aggregates anchored on nitrogen-doped graphene (Fe_2_O_3_/Fe_3_O_4_/NG) as an anode of SIBs. Upon a microwave plasma process, the Fe_2_O_3_/Fe_3_O_4_/NG composite exhibited rich phase boundaries and voids. When working as an SIB anode material, the Fe_2_O_3_/Fe_3_O_4_/NG shows high specific capacity (362 mAh g^−1^ at 100 mA g^−1^), excellent cycling stability (84% capacity retention after 100 cycles at 1 A g^−1^), and superior high-rate capability. The improved electrochemical performances are due to fast electron transport through Fe_3_O_4_, accelerated Na^+^ ion transport through Fe_2_O_3_/Fe_3_O_4_ phase boundaries, and voids in the nano-aggregates.

## 2. Materials and Methods 

### 2.1. Materials Preparation

The graphene oxide (GO) used in this work was produced from natural graphite flakes (Acros, Geel, Belgium) by a modified Hummer’s method [21]. The aqueous GO dispersion was centrifuged at 11,000 rpm for 30 min and was redispersed in de-ionized (DI) water more than 3 times. By discarding the supernatant, GO suspension in N, N-dimethyl formamide (DMF, 98%, Sigma-Aldrich, St. Louis, MO, USA) with 2 mg mL^−1^ was made.

Fe_2_O_3_/NG was synthesized by a solvothermal method. Fe(NO_3_)_3_·9H_2_O (840 mg, 99.9%, Sigma-Aldrich) was dissolved in the as-obtained GO suspension (100 mL). After stirring for 60 min, the mix was transferred into a Teflon-lined autoclave and was kept at 180 °C for 6 h. The as-obtained gel was rinsed in DI water for 24 h, then freeze-dried at −60 °C, 20 Pa for 24 h to generate Fe_2_O_3_/NG. The as-prepared bulk Fe_2_O_3_/NG was ground to powder with a mortar and pestle. After treating in a home-made microwave plasma fluidized bed (power of microwave = 400 W, Ar 40 sccm, H_2_ 10 sccm, 20 min), Fe_2_O_3_/Fe_3_O_4/_NG was obtained.

### 2.2. Materials Characterization

The crystal structure and phases of the material were characterized by X-ray diffraction (XRD, Bruker AXS GmbH, Karlsruhe, Germany) using Cu-Kα radiation. Morphology was analyzed by a field emission scanning electron microscope (SEM, Hitachi S4800, Tokyo, Japan). The transmission electron microscope (TEM) and high-resolution transmission electron microscope (HRTEM) were performed on a Hitachi HT7700 (Tokyo, Japan) and Talos F200X (Waltham, MA, USA), respectively. Thermogravimetric analysis was performed on PE STA8000 (Waltham, MA, USA). X-ray photoelectron spectroscopy (XPS) was performed on KRATOS Axis Supra XPS system (Kratos, Manchester, UK). The measured binding energies were calibrated to the reference energy by C1s = 284.6 eV. Raman spectroscopy was measured using the RM2000 system (Renishaw, London, UK), using a laser source with 17 mW at 532 nm.

### 2.3. Electrochemical Measurements

The active material, carbon black, and polyvinylidene fluoride (PVDF, Sigma-Aldrich) were added to N-methyl-2-pyrrolidone (NMP, 98%, Sigma-Aldrich) at a mass ratio of 8: 1: 1, and fully stirred to obtain a slurry. The resulting slurry was applied to copper foil, and dried in a vacuum oven at 100 °C for 12 h to obtain negative electrodes. The total mass loadings on a copper foil (20 mm in diameter, 99.99%, Sigma-Aldrich) are 1.13 and 0.75 mg for the Fe_2_O_3_/Fe_3_O_4_/NG and Fe_2_O_3_/NG electrodes, respectively. They were electrochemically characterized in CR2016-type coin cells, using Na metal as the counter electrode and reference electrode, and glass microfiber (Whatman, Little Chalfont, UK) as the separator. Dissolving 1 M NaClO_4_ (98%, Acros) in a mixed solvent of ethylene carbonate (EC, Acros) and diethyl carbonate (DEC, Acros) with 1:1 volume ratio, and adding 5 vol.% of fluoroethylene carbonate (FEC, Acros) additive, the electrolyte was prepared. Galvanostatic charge-discharge were performed on a LAND battery tester (Wuhan, China) in range of 0.01 to 3.0 V (vs. Na/Na^+^). Cyclic voltammetry (CV) and electrochemical impedance spectroscopy (EIS) measurements were performed using the CHI660E electrochemical workstation (Shanghai, China). All as-assembled half cells stood in a glove box for 12 h EIS tests without cycling. All capacities were calculated based on the mass of the active materials.

## 3. Results

**Scheme 1:**
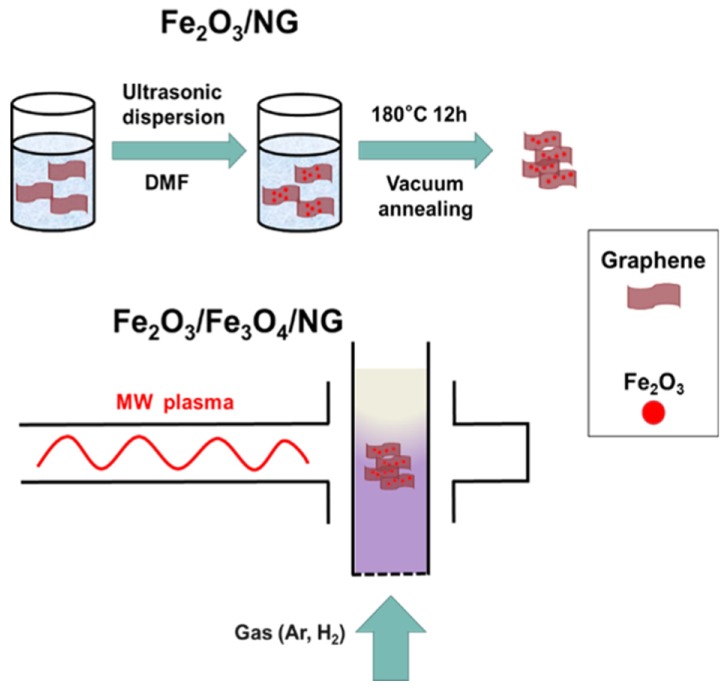
Schematics of synthesis of Fe_2_O_3_/NG and Fe_2_O_3_/Fe_3_O_4_/NG composites.

The Fe_2_O_3_/Fe_3_O_4_/NG nanocomposite was obtained by a solvothermal synthesis of Fe_2_O_3_/NG followed by a microwave plasma treatment. Taking advantage of the activation ability of plasma, the Fe_2_O_3_ was partially reduced to the Fe_3_O_4_, and Fe_2_O_3_/Fe_3_O_4_ nanoaggregates were formed, as illustrated in Scheme 1 [22].

**Figure 1:**
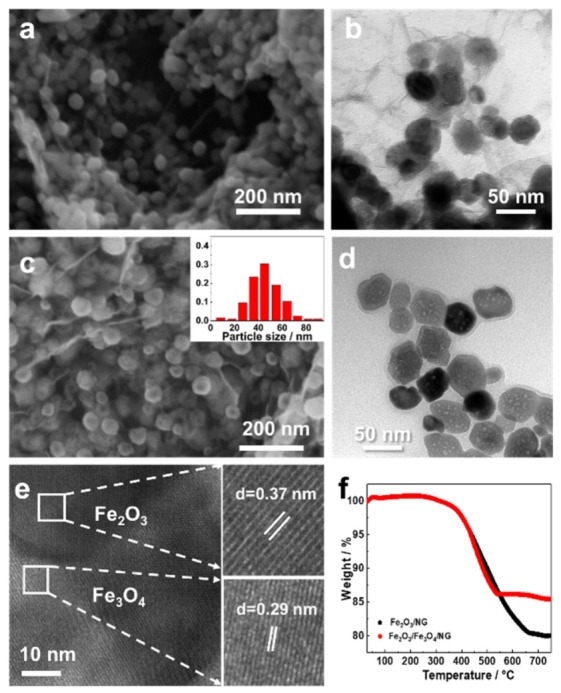
(**a**,**b**) SEM, TEM images of Fe_2_O_3_/NG composites. (**c**,**d**) SEM, TEM images of Fe_2_O_3_/Fe_3_O_4_/NG composites. The inset shows particle size distribution of Fe_2_O_3_/Fe_3_O_4_. (**e**) An HRTEM image of a Fe_2_O_3_/Fe_3_O_4_ particle, the right two panels denote interplane spacings of Fe_2_O_3_ and Fe_3_O_4_. (**f**) TGA curves of Fe_2_O_3_/NG and Fe_2_O_3_/Fe_3_O_4_/NG composites.

As shown in the SEM and TEM images (Figure 1a,b), Fe_2_O_3_ nanoparticles with size around 50 nm are well enveloped in graphene in the Fe_2_O_3_/NG composite. After plasma radiation, the morphology of the composite and the particle size distribution showed no significant change (Figure 1c and the inset). Under TEM observation, however, the previous spherical or ellipsoidal particles turned out to be in irregular shape. Meanwhile, dark and bright areas can be differentiated, of which the bright area is perhaps voids in the particles (Figure 1d). HRTEM reveals that the inhomogeneity of the particles stems from phase segregation of Fe_2_O_3_ and Fe_3_O_4_. In a close view of a particle, fringe spacing of 0.37 nm (corresponding to (012) plane of Fe_2_O_3_) and 0.29 nm (corresponding to the (220) plane of Fe_3_O_4_) can be found simultaneously (Figure 1e). The results confirmed that the Fe_2_O_3_ was partially reduced to Fe_3_O_4_ by energetic species in the plasma. Driven by phase segregation, Fe_2_O_3_/Fe_3_O_4_ nano-aggregates were eventually formed. The mass fraction of active materials ise about 87 and 80 wt.% for Fe_2_O_3_/Fe_3_O_4_/NG and Fe_2_O_3_/NG, respectively, as calculated from the thermal-gravimetric analysis and differential thermal analysis (TGA/DTA) results (Figure 1f).

**Figure 2:**
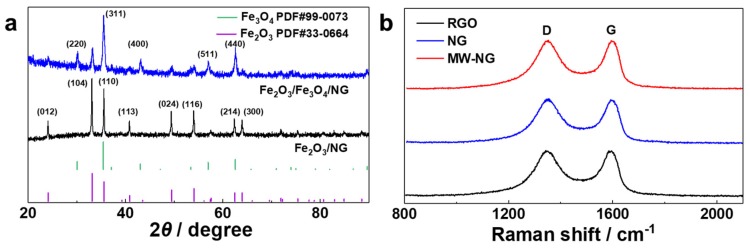
(**a**) XRD patterns of Fe_2_O_3_/Fe_3_O_4_/NG and Fe_2_O_3_/NG composites. (**b**) Raman spectra of microwave processed NG (MW-NG), NG, and RGO in the range of 1000–1800 cm^−1^.

XRD analysis shows diffraction peaks at 24.13, 33.15, 35.61, 40.85, 49.48, 54.09, 62.45, and 63.9° for the Fe_2_O_3_/NG (Figure 2a), which are corresponding to the (012), (104), (110), (113), (024), (116), (214), and (300) planes of Fe_2_O_3_ (PDF No.33-0664), respectively. For the Fe_2_O_3_/Fe_3_O_4_/NG, additional peaks at 30.07, 37.05, and 47.13° can be found, which are corresponding to the (220), (222), and (331) planes of Fe_3_O_4_ (PDF No 99-0073), respectively, confirming coexistence of Fe_2_O_3_ and Fe_3_O_4_ phases (Figure 2a). The Raman D peak of RGO are upshifted from 1340 (RGO) to 1343 cm^−1^ (NG) and G peak from 1573 to 1576 cm^−1^ after N doping (Figure 2b). This may be due to the strain of the graphene basal plane caused by C–N bond, indicating the successful insertion of nitrogen heteroatoms [23]. Additionally, the unchanged Raman spectrum after microwave plasma process indicates stable N doping upon plasma (red curve in Figure 2b).

**Figure 3:**
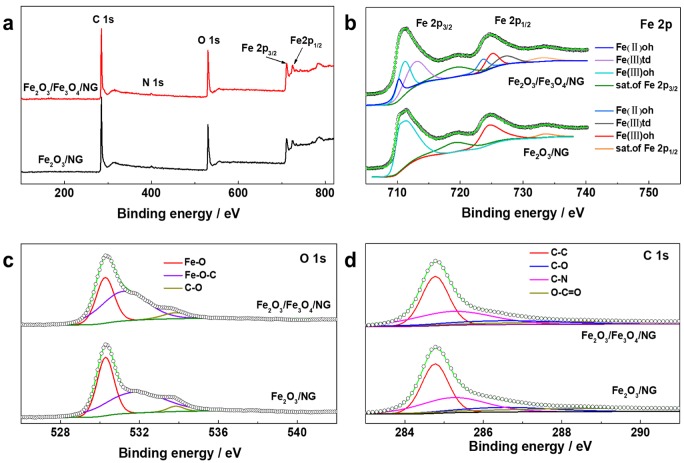
(**a**) XPS spectra of Fe_2_O_3_/Fe_3_O_4_/NG and Fe_2_O_3_/NG. The corresponding (**b**) Fe 2p spectra, (**c**) O 1s spectra, and (**d**) C 1s spectra.

XPS survey scans discovered Fe 2p, O 1s, N 1s, and C 1s states in both Fe_2_O_3_/Fe_3_O_4_/NG and Fe_2_O_3_/NG (Figure 3a). In Figure 3b, the high-resolution Fe 2p spectra of the composites have two main peaks at 711.2 and 724.7 eV, corresponding to Fe 2p_3/2_ and Fe 2p_1/2_, respectively. In the Fe_2_O_3_/NG, the Fe 2p spectrum consists of a series of characteristic peaks of Fe^3+^ in Fe_2_O_3_, which are two spin energy separated components (Fe^3+^ (oh) at 711.1 and 725.1 eV), Fe 2p_3/2_ satellite (719.7 eV), and Fe 2p_1/2_ satellite (733.8 eV) signals, but no F^0^e or Fe^2+^ signal is observed. The Fe_2_O_3_/Fe_3_O_4_/NG has not only the same components with Fe_2_O_3_/NG, but also Fe^2+^ (oh), Fe^3+^(td), Fe^2+^(oh), and Fe^3+^(td) signals of Fe_3_O_4_ at 710.0, 713.0, 723.6, and 727.3 eV, respectively [17]. The XPS analysis clearly evidences the coexistence of Fe_2_O_3_ and Fe_3_O_4_ in the hybrid material. With the component area ratio of Fe^2+^ to Fe^3+^, the atomic ratio of Fe^2+^ to Fe^3+^ can be estimated to be 0.102. Combined with the TGA results, the mass loading of Fe_2_O_3_ and Fe_3_O_4_ are calculated to be 0.61 and 0.23 mg, respectively, as described in the Supporting Information for detail. 

As shown in Figure 3c, the deconvolution of the O 1s profiles reveals three components, Fe–O (530.2 eV), Fe–O–C (531.2 eV), and C–O (533.8 eV). The areal percentage of the Fe–O–C bonds component increases from 53.5% for the Fe_2_O_3_/NG to 58% for the Fe_2_O_3_/Fe_3_O_4_/NG. The additional Fe–O–C bonds can not only promote the electron transport in the Fe_2_O_3_/Fe_3_O_4_/NG, but also stabilize the Fe_2_O_3_/Fe_3_O_4_ on the graphene sheet during charge/discharge, thereby improving the electrochemical kinetics and stability. [24] In Figure 3d, deconvolution of the C 1s profiles of the nanostructured hybrid material reveals four components including C–C (284.7 eV), C–N (285.2 eV), C–O(286.5 eV), and O–C=O (289.35 eV) [25,26], which remain unchanged before and after the plasma process, except for a slight areal ratio decrease of the O–C=O bonds from 22.78% for the Fe_2_O_3_/NG to 16.64% for the Fe_2_O_3_/Fe_3_O_4_/NG.

**Figure 4:**
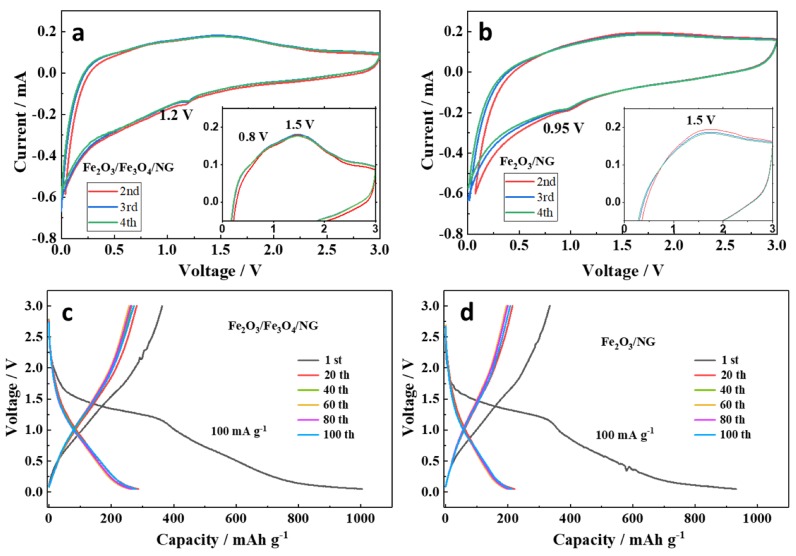
CV curves of (**a**) Fe_2_O_3_/Fe_3_O_4_/NG and (**b**) Fe_2_O_3_/NG anodes from the 2nd to 4th cycle at a scan rate of 1.0 mV s^−1^ between 0.05 and 3 V vs. Na/Na^+^. Galvanostatic charge-discharge curves of (**c**) Fe_2_O_3_/Fe_3_O_4_/NG and (**d**) Fe_2_O_3_/NG anodes at a current density of 100 mA g^−1^.

The CV curves from the 2nd to the 4th cycle of the electrodes are shown in Figure 4a,b. In the cathodic sweeps, a peak around 1.2 V for the Fe_2_O_3_/Fe_3_O_4_/NG in Figure 4a can be assigned to the reduction of Fe^2+^/Fe^3+^ to Fe^0^ (Equations (1) and (2)), while the peak around 0.9 V for the Fe_2_O_3_/NG in Figure 4b corresponds to the reduction of Fe^3+^ to Fe^0^ (Equation (1)). In the anodic sweeps, broad anodic peaks around 1.5 V are ascribed to the oxidation of Fe^0^ to Fe^3+^ for both electrodes (insets in Figure 4a,b) [24,27]. Only the Fe_2_O_3_/Fe_3_O_4_/NG electrode has a small shoulder peak at 0.8 V (inset in Figure 4a), which is an indication of the oxidization of Fe^0^ to Fe^2+^ [19]. The lower peak-to-peak separation between the redox peaks and better coincidence of the sweeps of the Fe_2_O_3_/Fe_3_O_4_/NG suggest faster charge transfer kinetics and better reversibility.

Fe_2_O_3_ + 6 Na^+^ + 6 e^−^ ⟷ 2 Fe^0^ + 3 Na_2_O (1)

Fe_3_O_4_ + 8 Na^+^ + 8 e^−^ ⟷ 3 Fe^0^ + 4 Na_2_O (2)

Figure 4c,d shows charge–discharge curves of the two types of electrodes at a current density of 100 mA g^−1^ in the voltage window of 0.05–3.0 V (vs. Na/Na^+^). The initial specific discharge and charge capacities of the Fe_2_O_3_/NG electrode are 931 and 334 mAh g^−1^, respectively, with an initial coulombic efficiency of 36%. After the microwave plasma process, the obtained Fe_2_O_3_/Fe_3_O_4_/NG composite exhibits initial specific discharge capacity of 1004 mAh g^−1^, charge capacity of 363 mAh g^−1^, and an almost identical initial coulombic efficiency. The lost capacity mainly stems from the formation of SEI and probably degradation of the electrolyte. In the subsequent discharge–charge cycles, the Fe_2_O_3_/Fe_3_O_4_/NG electrode has significantly higher capacities than the Fe_2_O_3_/NG. 

**Figure 5:**
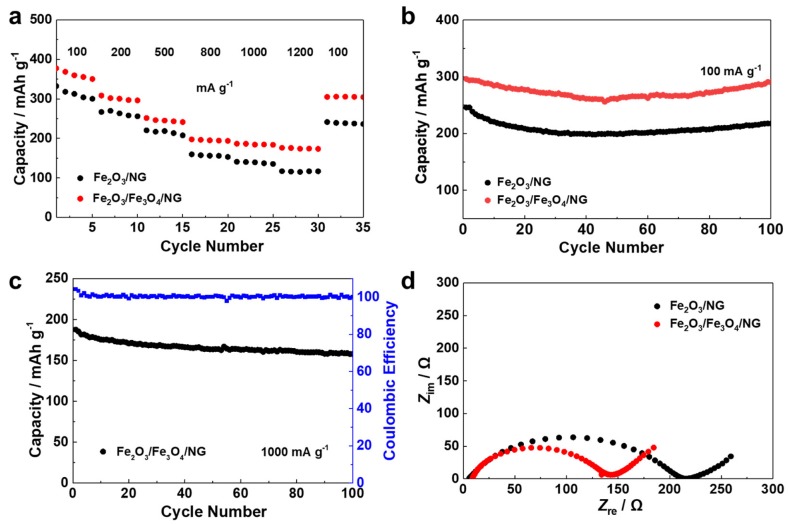
(**a**) Rate performance of Fe_2_O_3_/NG and Fe_2_O_3_/Fe_3_O_4_/NG tested at current densities from 100 to 1200 mA g^−1^. (**b**) Cycling performances of Fe_2_O_3_/NG and Fe_2_O_3_/Fe_3_O_4_/NG at current density of 100 mA g^−1^. (**c**) Cycling performance of Fe_2_O_3_/Fe_3_O_4_/NG at current density of 1000 mA g^−1^. (**d**) Nyquist plots of electrochemical impedance spectra (EIS) of Fe_2_O_3_/NG and Fe_2_O_3_/Fe_3_O_4_/NG.

The rate performances of Fe_2_O_3_/NG and Fe_2_O_3_/Fe_3_O_4_/NG electrodes are compared in Figure 5a. With progressively growing current density from 100 to 1200 mA g^−1^, the Fe_2_O_3_/Fe_3_O_4_/NG composite constantly exhibits higher discharge capacities than the Fe_2_O_3_/NG. After a series of cycles, a reversible discharge capacity of 305 mAh g^−1^ at 100 mA g^−1^ is reached for the Fe_2_O_3_/Fe_3_O_4_/NG, comparing with 239 mAh g^−1^ for the Fe_2_O_3_/NG. The specific capacity contributed by Fe_2_O_3_ in the Fe_2_O_3_/Fe_3_O_4_/NG can be estimated by the XPS and TGA results. For example, the reversible capacity contributed by Fe_2_O_3_ after the rate performance testing at 100 mA g^−1^ can be 307–358 mAh g^−1^, which is higher than the 239 mAh g^−1^ for the Fe_2_O_3_/NG. This evidently suggests a synergetic effect of Fe_2_O_3_ and Fe_3_O_4_ in the electrode. The calculation of capacity contributed by Fe_2_O_3_ is detailed in the Supporting Information.

In Figure 5b, the cycling performance of Fe_2_O_3_/NG and Fe_2_O_3_/Fe_3_O_4_/NG electrodes in 100 cycles are compared at a current density of 100 mA g^−1^. After 100 cycles, the capacity of Fe_2_O_3_/Fe_3_O_4_/NG is 291 mAh g^−1^, which is much higher than that of Fe_2_O_3_/NG 218 mAh g^−1^. At a higher current density (1000 mA g^−1^), Fe_2_O_3_/Fe_3_O_4_/NG can still discharge 158 mAh g^−1^ after 100 cycles (capacity retention rate 84%, Figure 5c).

In Figure 5d, the electrochemical impedance spectra (EIS) Nyquist plots of Fe_2_O_3_/NG and Fe_2_O_3_/Fe_3_O_4_/NG electrodes present a depressed semicircle in the high-frequency region and a straight line in the low-frequency region. To extract the EIS parameters, an equivalent circuit is proposed, as displayed in Supplementary Figure S1. The EIS tests were performed before cycling of the electrodes, with open circuity voltages of 1.65 and 1.47 V for the Fe_2_O_3_/NG and Fe_2_O_3_/Fe_3_O_4_/NG, respectively. As listed in Table 1, both cells present similar values of electrolyte resistance (*R*_s_). The Fe_2_O_3_/Fe_3_O_4_/NG electrode has a charge transfer resistance (*R*_ct_: 134.6 Ω) that is only half of the Fe_2_O_3_/NG (210.7 Ω). The reduction of the semicircle (or *R*_ct_) is due to more Fe-O-C bonds and the appearance of Fe_3_O_4_ in the electrode, indicating that the charge transfer kinetics in Fe_2_O_3_/Fe_3_O_4_/NG is faster. Moreover, the one order of magnitude higher diffusion coefficient of Na^+^ (*D*_Na^+^_, obtained by fitting the EIS, see Supporting Information for detail) in the Fe_2_O_3_/Fe_3_O_4_/NG can be attributed to the fast Na^+^ diffusion through the phase boundaries and voids in the Fe_2_O_3_/Fe_3_O_4_ heterostructure.

**Figure 6:**
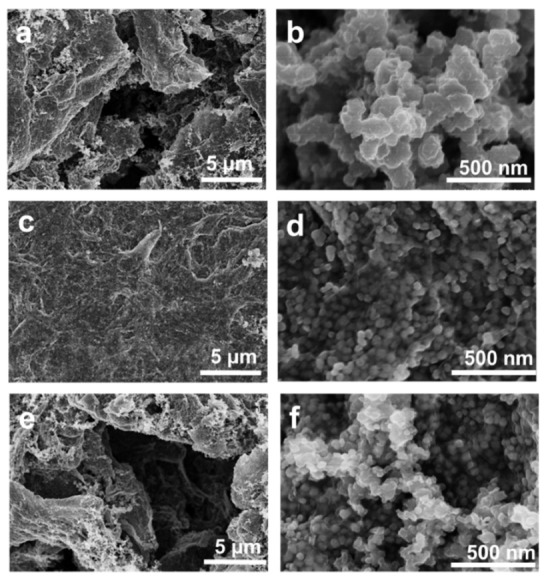
SEM images of the (**a**,**b**) Fe_2_O_3_/NG and (**c**,**d**) Fe_2_O_3_/Fe_3_O_4_/NG electrodes after 100 discharge-charge cycles at 1000 mA g^−1^ at different magnifications. SEM images of the (**e**,**f**) Fe_2_O_3_/Fe_3_O_4_/NG electrodes after 300 discharge–charge cycles at 1000 mA g^−1^ at different magnifications.

The long-term cycling performance is elucidated by post-mortem observation, as shown in Figure 6. Serious cracks and particle swelling can be observed for the Fe_2_O_3_/NG electrode after 100 discharge-charge cycles at 1000 mA g^−1^ (Figure 6a,b). By contrast, in the same condition, the Fe_2_O_3_/Fe_3_O_4_/NG electrode remained almost unchanged in terms of particle shape and size (Figure 6c,d). In a word, the comparison clearly demonstrates the stabilizing effect of the Fe_2_O_3_/Fe_3_O_4_/NG electrodes structure. Even after 300 cycles, the Fe_2_O_3_/Fe_3_O_4_/NG electrode could retain its particle size, although cracks occurred (Figure 6e,f).

## 4. Discussion

**Scheme 2:**
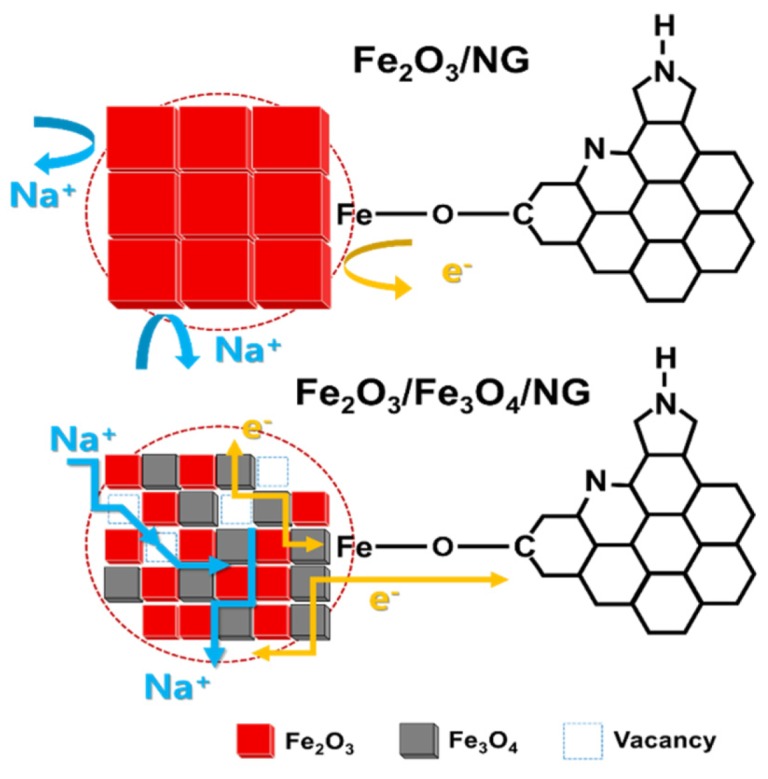
Schematics of structures, electron transfer and transport, and ion diffusion in Fe_2_O_3_/NG (upper) and Fe_2_O_3_/Fe_3_O_4_/NG (lower) composites.

The outstanding sodium storage properties of the Fe_2_O_3_/Fe_3_O_4_/NG can be attributed to its unique structural features, as illustrated in Scheme 2. First, the highly conductive Fe_3_O_4_ improves electron transport in the hybrid Fe_2_O_3_/Fe_3_O_4_ nano-aggregates. Second, the phase boundaries and voids in the Fe_2_O_3_/Fe_3_O_4_ heterostructure provide fast diffusion channels for Na^+^ ions. Third, the robust interfacial interaction reinforced by Fe–O–C bonds can not only maintain the integrity of the electrode during the long-term cycles, but also provide a highway for electron transfer between the graphene and the Fe_2_O_3_/Fe_3_O_4_ nano-aggregates. Moreover, N-doping also assists the sodium storage, increasing the reversible capacity of materials. All these structural features endowed by the microwave plasma process lead to outstanding reversible capacity, good rate performance, and long-term cycling stability. As compared in Table 2, the electrochemical performance of the Fe_2_O_3_/Fe_3_O_4_/NG is among the top-ranking of the reported iron oxide/graphene-based SIB anodes.

## 5. Conclusions

In summary, the Fe_2_O_3_/Fe_3_O_4_ nano-aggregates anchored on nitrogen-doped graphene as an anode for sodium-ion batteries were successfully prepared with the assistance of the microwave plasma process. The highly conductive Fe_3_O_4_ in the composite improves electron transport, and the voids and phase boundaries facilitate Na^+^ ion diffusion into the Fe_2_O_3_/Fe_3_O_4_ heterostructure. Moreover, the Fe–O–C bonds not only strength the structural robustness, but also electrically bridge the graphene and the Fe_2_O_3_/Fe_3_O_4_ nano-aggregates. Consequently, the Fe_2_O_3_/Fe_3_O_4_/NG anode exhibits outstanding electrochemical performance, i.e., high specific capacity, excellent rate capability, and stable long-term cycling performance. The design concept of this unique heterostructure can be extended to other energy storage applications based on metal oxides.

## Figures and Tables

**Table 1:** The fitted EIS parameters and diffusion coefficients of Fe_2_O_3_/NG and Fe_2_O_3_/Fe_3_O_4_/NG.

Electrode	*R_s_* (Ω)	*R_ct_* (Ω)	*D*_Na^+^_ (cm^2^ s^−1^)
Fe_2_O_3_/NG	6	210.6	1.65 × 10^−12^
Fe_2_O_3_/Fe_3_O_4_/NG	8.9	134.7	1.34 × 10^−11^

**Table 2:** Comparison of the specific capacities of iron oxide based anodes in literature and Fe_2_O_3_/Fe_3_O_4_/NG anode in this work.

Anodes	Current Density (mA g^−1^)				References
	Capacity (mAh g^−1^)				
Fe_2_O_3_/Fe_3_O_4_/NG	100	200	1000	1200	This work
	362	300	185	174	
Fe_2_O_3_/NG	50	100	200	1000	Meng 2017 [28]
	343	285	230	132	
Fe_2_O_3_/C	50	100	200	1000	Zhang 2018 [29]
	364	291	245	150	
Fe_3_O_4_/G	100	200	500	1000	Fu 2016 [30]
	310	225	180	140	
Fe_3_O_4_@C/G	100	200	500	1000	Qi 2019 [31]
	375	300	254	200	
Fe_2_O_3_@NC	200	500	1000	4000	Guo 2018 [32]
	289	253.7	221.5	167.8	
Fe_3_O_4_/G/QD	100	200	500	1000	Liu 2016 [33]
	316	273	216	113	
Fe_3_O_4_/C	100	200	500	1000	Wang 2017 [34]
	293	262	223	195	
Fe_3_O_4_@N–C	80	240	400	800	Zhao 2019 [35]
	386	315	277	248	

## Supplementary Materials

The following are available online at https://www.mdpi.com/2079-4991/10/4/782/s1, Figure S1: (a) the equivalent electrical circuit (b,c) The *Z*’-*ω^−^*^1/2^ plots for the Fe_2_O_3_/NG and Fe_2_O_3_/Fe_3_O_4_/NG. Table S1. Calculation of capacity contributed by Fe_2_O_3_ in the Fe_2_O_3_/Fe_3_O_4_/NG electrode.
