# Supplementary material for: Plasma Enabled Fe2O3/Fe3O4 Nano-aggregates Anchored on Nitrogen-doped Graphene as Anode for Sodium-Ion Batteries
Source: Nanomaterials (Basel). 2020 Apr 18;10(4):782. doi: 10.3390/nano10040782 (PMC7221635; doi:10.3390/nano10040782)
Supplement: Supplementary file 1 [file nanomaterials-10-00782-s001.pdf]

Supplementary Materials: Plasma Enabled Fe_2O_3/Fe_3O_4 Nano-aggregates Anchored on Nitrogen-doped Graphene as Anode for Sodium-ion Batteries

Qianqian Wang [1], Yujie Ma [1], Li Liu [1], Shuyue Yao [1], Wenjie Wu [1], Zhongyue Wang [1], Peng Lv [1], Jiajin Zheng [1], Kehan Yu [1,*], Wei Wei [1] and Kostya (Ken) Ostrikov [2,3]

[1] School of Electronic and Optical Engineering Nanjing University of Posts and Telecommunications, Nanjing 210023, P.R. China; wqq1242103532@163.com (Q.W.); 1017030916@njupt.edu.cn (Y.M.); a884205500701@icloud.com (L.L.); ysynn0916@163.com (S.Y.); wuvenjie@163.com (W.W.); zywang@njupt.edu.cn (Z.W.); lvp@njupt.edu.cn (P.L.); zhengjj@njupt.edu.cn (J.Z.); weiwei@njupt.edu.cn (W.W.)

[2] School of Chemistry and Physics, Queensland University of Technology, Brisbane QLD 4000, Australia; kostya.ostrikov@qut.edu.au

[3] CSIRO-QUT Joint Sustainable Processes and Devices Laboratory P.O. Box 218, Lindfield NSW 2070, Australia

* Correspondence: kehanyu@njupt.edu.cn

I. Estimation of capacity contributed by Fe_2O_3 in the Fe_2O_3/Fe_3O_4/NG electrode.

The ratio of Fe_2O_3 to Fe_3O_4 in the composite can be estimated by comparing the peak components of the Fe^{2+} and Fe^{3+} in the XPS. The components of Fe^{2+} include two Fe^{2+}(oh) peaks, the components of Fe^{3+} include Fe^{3+}(oh), Fe^{3+}(td), and two satellite peaks.

(1) First, the atomic ratio (R_1) of Fe^{2+} to Fe^{3+} can be calculated by the areas (A) of the component peaks: $A_1(Fe^{2+}(oh)) + A_2(Fe^{2+}(oh)) = 30361.6$; $A_1(Fe^{3+}(oh)) + A_2(Fe^{3+}(oh)) = 75050.9$; $A_1(Fe^{3+}(td)) + A_2(Fe^{3+}(td)) = 114755.8$; $A(\text{sat. 1}) = 83488.2$; $A(\text{sat. 2}) = 23423.8$, which are obtained by careful deconvolution of the peaks.

$$R_1 = \frac{A(\mathrm{Fe}^{2+})}{A(\mathrm{Fe}^{3+})}$$
$$= \frac{A_1(\mathrm{Fe}^{2+}(\mathrm{oh})) + A_2(\mathrm{Fe}^{2+}(\mathrm{oh}))}{A_1(\mathrm{Fe}^{3+}(\mathrm{oh})) + A_2(\mathrm{Fe}^{3+}(\mathrm{oh})) + A_1(\mathrm{Fe}^{3+}(\mathrm{td})) + A_2(\mathrm{Fe}^{3+}(\mathrm{td})) + A(\mathrm{sat.}\,1) + A(\mathrm{sat.}\,2)}$$
$$= 0.102$$

(2) Let the molar ratio of Fe_2O_3 to Fe_3O_4 is R_2,

$$R_2 = \frac{1}{2R_1} - 1 = 3.89$$

(3) The mass ratio of Fe_2O_3 to Fe_3O_4 in the Fe_2O_3/Fe_3O_4/NG composite is thus equal to $R_3 = 0.69 * R_2 = 2.68$.

The mass of Fe_2O_3 and Fe_3O_4 in the Fe_2O_3/Fe_3O_4/NG can thus be calculated with R_3 and the TGA result, in which shows a 13% weight loss. The weight loss is attribtuted to the oxidization

of burning out of graphene, carbon black, and binder, but the oxidzation of Fe_3O_4 should also be considered. It's easy to get the weight percentages of Fe_2O_3 and Fe_3O_4 in the Fe_2O_3/Fe_3O_4/NG composite are 59 wt.% and 22 wt.%, respectively. The mass of the Fe_2O_3/Fe_3O_4/NG composite in an electrode is 1.03 mg, then the masses of Fe_2O_3 and Fe_3O_4 are 0.61 and 0.23 mg, respectively

We assume the capacity of an electrode is contributed by the iron oxide, ignoring the capacity of graphene owing to low content. Then the capacity of an electrode is a sum of that of Fe_2O_3 and Fe_3O_4. Although we cannot figure out the actual capacity of Fe_3O_4, a capacity range can be given by literature search. The lower and upper bounds of Fe_3O_4 specific capacity is obtained in [Fu et al. *RSC Adv.*, 2016, 6, 16624] and [Qi et al. *ChemistrySelect* 2019, 4, 2668], in which the Fe_3O_4 nano particles are also in the Fe_3O_4/graphene circumstances. Now, the upper and lower bounds of specific capacity of Fe_2O_3 in Fe_2O_3/Fe_3O_4/NG can be calculated accordingly, as listed in Table S1. Note that even the lower bound of the specific capacity of Fe_2O_3 in Fe_2O_3/Fe_3O_4/NG is higher than that in Fe_2O_3/NG, which is the evidence of the synergic effect of Fe_2O_3/Fe_3O_4 in the electrode.

Table S1. Calculation of capacity contributed by Fe_2O_3 in the Fe_2O_3/Fe_3O_4/NG electrode.

Current density (mA g^{-1})	Capacity of Fe_2O_3/Fe_3O_4/NG (mAh)	Specific capacity of Fe_3O_4 in literature (mAh g^{-1})		Specific capacity of Fe_2O_3 in Fe_2O_3/Fe_3O_4/NG (mAh g^{-1})		Specific capacity of Fe_2O_3 in Fe_2O_3/NG (mAh g^{-1})
		Lower bound *	Upper bound **	Lower bound	Upper bound	
100	0.326	316	337	410	418	314
200	0.271	220	298	334	363	263
500	0.221	180	254	268	296	216
1000	0.167	140	200	199	222	147
100	0.275	250	386	307	358	239

* Fu et al. *RSC Adv.*, 2016, 6, 16624, https://doi.org/10.1039/C5RA25835A.

** Qi et al. *ChemistrySelect* 2019, 4, 2668, https://doi.org/10.1002/slct.201900663

II. The equivalent electrical circuit used for fitting the EIS data and diffusion coefficient of Na^+ ion in electrodes.

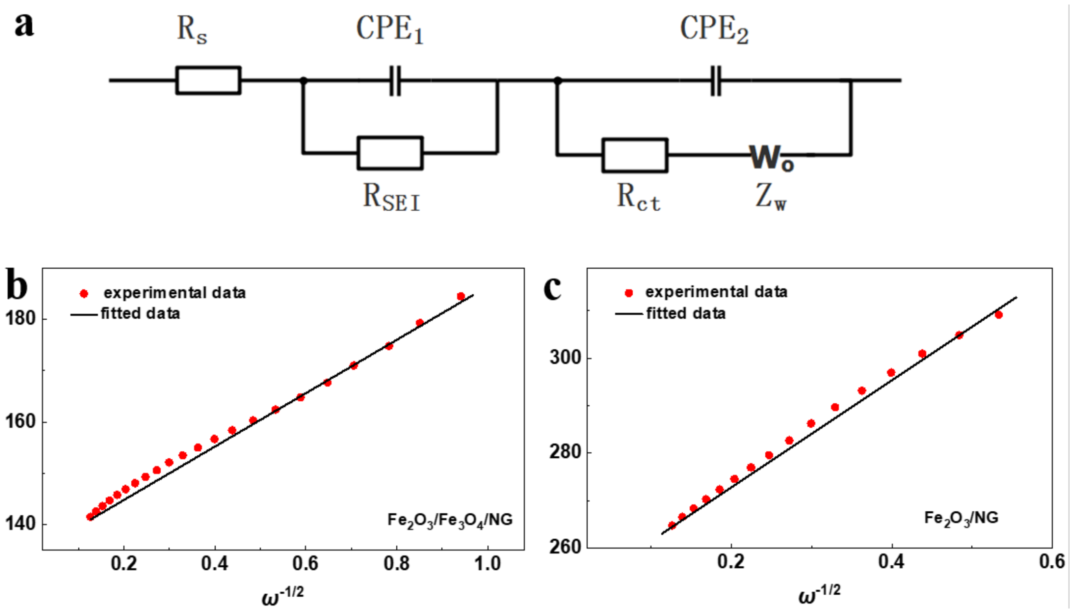

Figure S1. (a) The equivalent electrical circuit (b,c) The Z' - $\omega^{1/2}$ plots for the Fe_2O_3/NG and Fe_2O_3/Fe_3O_4/NG.

The sodium ion diffusion coefficient can be calculated from the formula as following:

$$D_{(Na+)} = \frac{R^2T^2}{2A^2n^4F^4C_{Na}^2\sigma^2} \qquad (1)$$

$$Z' = R_s + R_{ct} + \sigma^2\omega^{-1/2} \qquad (2)$$

in which the Reaction 3 includes surface area of the electrode(A), the number of the electrons per molecule attending the electronic transfer reaction(n), the Faraday constant (F), the concentration of sodiumion (C), the gas constant (R) , the room temperature in our experiment (T), the slope of the line Z' - $\omega^{1/2}$ (σ), respectively. where the constant values of F and R are 96500 C mol^{-1} and 8.314 $JK^{-1}mol^{-1}$, respectively. A is the electrode area which is 2 cm^2, n is 1, C can be calculated from the density and the molecular weight of the materials synthesized by different methods, which are 6.3×10^4 mol m^{-3}. In Eq. (2), R_s and R_{ct} are fitted parameters of Nyquist plots, and thus, σ can be calculated by the linear fitting between the real component (Z') of the impedance and $\omega^{1/2}$ (ω: angular frequency). According to the fitted slope of Z' - $\omega^{1/2}$ observed in SIB (see Figure S2b, c), the ionic diffusion coefficient of Fe_2O_3/Fe_3O_4/NG (1.34×10^{-11} cm^2s^{-1}) is bigger than that of Fe_2O_3/NG (1.65×10^{-12} cm^2s^{-1}).
